# Quantitative 3D investigation of Neuronal network in mouse spinal cord model

**DOI:** 10.1038/srep41054

**Published:** 2017-01-23

**Authors:** I. Bukreeva, G. Campi, M. Fratini, R. Spanò, D. Bucci, G. Battaglia, F. Giove, A. Bravin, A. Uccelli, C. Venturi, M. Mastrogiacomo, A. Cedola

**Affiliations:** 1 Institute of Nanotechnology-CNR c/o Physics Department at ‘Sapienza’ University, Piazzale Aldo Moro 2, 00185 Rome, Italy; 2 Institute of Crystallography-CNR, 00015 Monterotondo, Rome, Italy; 3 Fondazione Santa Lucia I.R.C.C.S., Via Ardeatina 306, 00179 Roma, Italy; 4 Department of Experimental Medicine, University of Genova & AUO San Martino - IST Istituto Nazionale per la Ricerca sul Cancro, Largo R. Benzi 10, 16132 Genova, Italy; 5 I.R.C.C.S. Neuromed, Località Camerelle, 86077 Pozzilli, Italy; 6 Museo Storico della Fisica e Centro Studi e Ricerche Enrico Fermi, Piazza del Viminale 1, 00184 Roma, Italy; 7 European Synchrotron Radiation Facility, 71 Avenue des Martyrs, 38043 Grenoble, Cedex France; 8 University of Genova DINOGMI Largo Daneo, 3 IT-16132 Genova, Italy; 9 IRCCS Azienda Ospedaliera Universitaria San Martino – IST, Genoa, Italy

## Abstract

The investigation of the neuronal network in mouse spinal cord models represents the basis for the research on neurodegenerative diseases. In this framework, the quantitative analysis of the single elements in different districts is a crucial task. However, conventional 3D imaging techniques do not have enough spatial resolution and contrast to allow for a quantitative investigation of the neuronal network. Exploiting the high coherence and the high flux of synchrotron sources, X-ray Phase-Contrast multiscale-Tomography allows for the 3D investigation of the neuronal microanatomy without any aggressive sample preparation or sectioning. We investigated healthy-mouse neuronal architecture by imaging the 3D distribution of the neuronal-network with a spatial resolution of 640 nm. The high quality of the obtained images enables a quantitative study of the neuronal structure on a subject-by-subject basis. We developed and applied a spatial statistical analysis on the motor neurons to obtain quantitative information on their 3D arrangement in the healthy-mice spinal cord. Then, we compared the obtained results with a mouse model of multiple sclerosis. Our approach paves the way to the creation of a “database” for the characterization of the neuronal network main features for a comparative investigation of neurodegenerative diseases and therapies.

The investigation of neurodegenerative diseases, immune-mediated central nervous system (CNS) diseases and the monitoring of effective therapies for neurological diseases are of fundamental importance, and the subject of an intense research activity.

Disorders of CNS that, like Alzheimer’s disease, Lewy body dementia and Parkinson’s disease include changes in neurite morphology, dramatic synapse and dendritic spine loss^1^, eventually, or like multiple sclerosis (MS) or amyotrophic lateral sclerosis (ALS)^2^,^3^, affect hundreds of millions of humans in the world. The pathophysiology of many of these diseases is poorly understood, and we still lack effective therapies.

In this framework, comparative pre-clinical investigations would require a ‘database’ of the parameters identifying a healthy neuronal network architecture, such as size, neuron density, neuron spatial distribution and correlation. Indeed, changes of these features in any part of the CNS would have important consequences for their efferent and afferent connections.

In order to build such a structural ‘database’ of the neuronal network architecture, an improvement in the performance of 3D imaging tools at high spatial resolution is required.

Multiple technologies^4^,^5^,^6^,^7^,^8^ have been used to understand the 3D morphology of individual neurons, glia and axons within the brain and the spinal cord. However, up to now, little progress has been made on understanding the arrangement of neurons in 3D space and the functional role of this arrangement, due to the limited field of view and/or low spatial resolution of currently available imaging tools. Recently, M. Fratini *et al*.^9^ overcame these limitations, using high-resolution X-ray Phase-Contrast multiscale-Tomography (XPCmT) to simultaneously image the 3D distribution of the small capillary network and of the neuronal network in an entire mouse spinal cord, covering a spatial range from millimeters to hundreds of nanometers. XPCmT does not require neither aggressive sample preparation nor sectioning that could compromise the results. However, the work of M. Fratini *et al*.^9^ provided only a qualitative description of the neuronal microanatomy.

Nevertheless, the attainment of quantitative information on the density and spatial distribution of the neurons within the CNS is expected to be of paramount importance for the tissue physiology.

In the literature, several mathematical approaches have been used to quantify the neuronal patterning in 2D^10^,^11^,^12^ and recently for neuroanatomical structures in 3D space^13^,^14^. The use of statistical analysis for the investigation of neuronal positioning has been explored extensively in the retina, where some classes of neurons are located with nearly crystalline order^15^,^16^,^17^,^18^. However, statistical analysis has not been applied to the CNS yet.

In the present work, firstly we show the healthy-mouse neuronal architecture in the spinal cord by imaging the 3D neuronal network using XPCmT with high spatial resolution and large field of view. Thanks to the high quality of the resulting images, we were able to perform a detailed quantitative analysis of the neuronal network. In particular, spatial statistical analysis was employed to obtain quantitative information about motor neurons arrangement at different levels of the spinal cord. To this end, we defined the following parameters, to effectively characterize the neurons spatial distribution:

1. *Clustering length and degree*, that tell us whether the neurons are aggregated (clustering) or dispersed (anti-clustering) in comparison to a Complete State of Randomness described by a homogenous Poisson process;
2. A *regularity factor*, given by the Voronoi tessellation, describing whether neurons are located in a more or less uniform way^12^.

Since these characteristic parameters of the neuronal microanatomy are expected to change in pathological conditions, we applied the spatial statistical analysis to detect and quantitatively characterize the modification of the motor neurons networks in an experimental autoimmune encephalomyelitis (EAE) mice model.

## Results

### Neuronal structure and morphology

**Figure 1:**
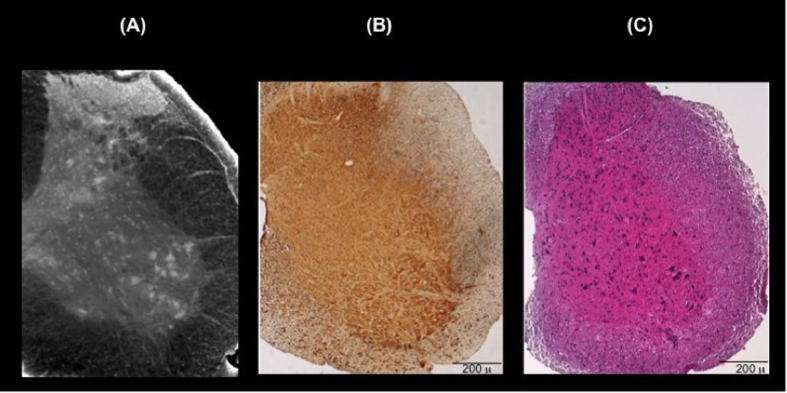
(**A**) 3D reconstruction of ~200 μm thick volume of the cervical region of a representative healthy mouse spinal cord. (**B**) Immunohistochemical analysis of SMI-32, a marker of motor neurons, and (**C**) hematoxylin/eosin staining in the cervical region of a representative healthy mouse spinal cord to confirm the 3D reconstruction of imaged spinal cord.

**Figure 2:**
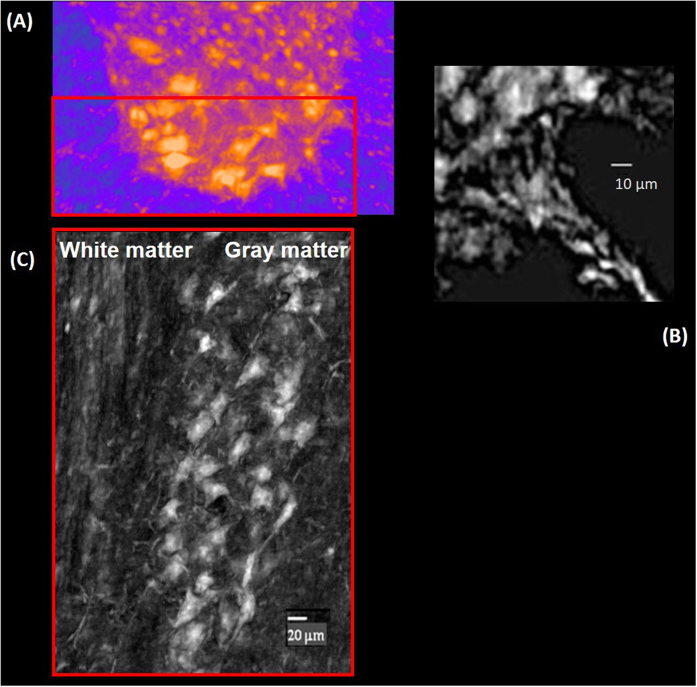
(**A**) Axial cross section of the spinal cord ventral horn in the thoracic level. The image was segmented to show the neurons (dark yellow) and the neuron fibers (light violet). (**B**) Motor nerve fiber is imaged at the interface between the gray and white matter in the red squared selected in (**A**). (**C**) 500 μm thick volume in sagittal view, of the spinal cord relative to the selected ROI in (**A**) (red box).

**Figure 3:**
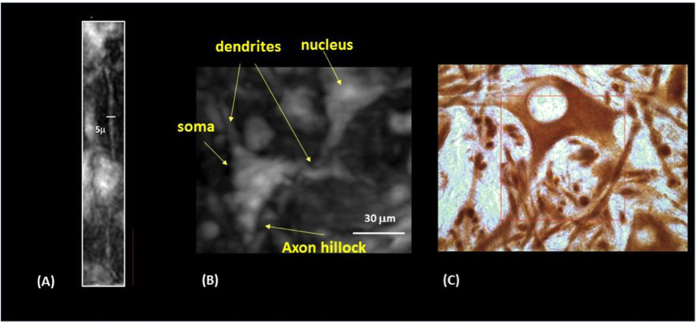
(**A**) A magnified image of one neuron fiber connection selected in a motor nucleus. (**B**) A magnification of the interaction between two motor neurons in the red box in Fig. 2A. (**C**) High magnification of a SMI-32-positive neuron to parallel the imaged neuron (red square 50 × 50 μm^2^).

**Figure 4:**
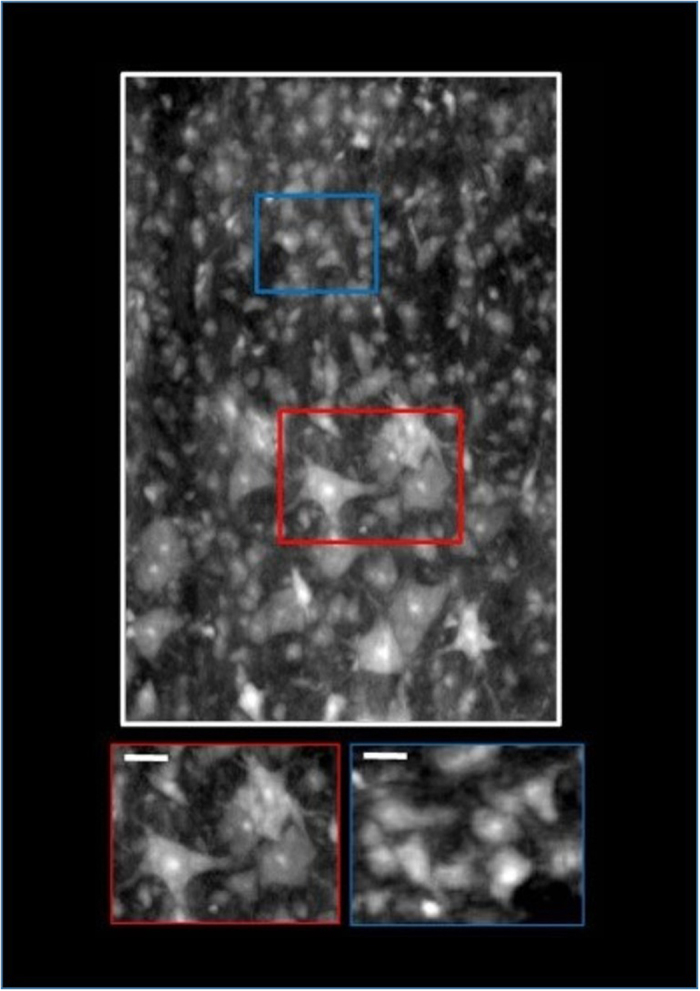
Longitudinal distribution of the cells, at the interface between the central region and the ventral horn crossing the motor neurons (red box), and smaller cells compatible with interneurons (blue box) and glia cells. The white scale bars in the insets are of 20 μm size.

**Figure 5:**
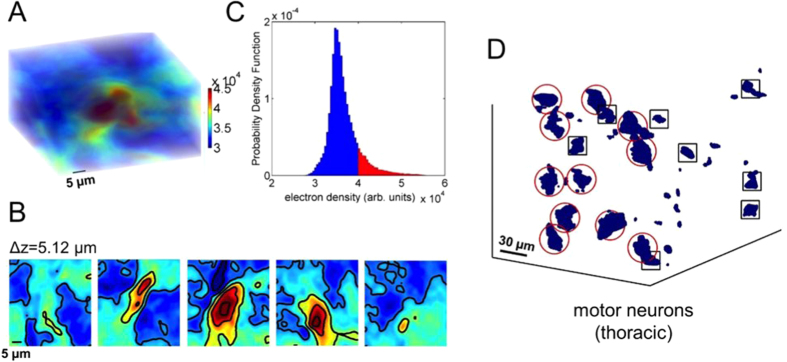
(**A**) Rendering of the electronic density of a typical single motor neuron in the thoracic region. **(B)** The contour plots identify the nucleus (region with higher intensity) in the (**A**) slabs sectioned at different levels. Each slice corresponds to a high of 5.12 μm. **(C)** The Probability density distribution of the motor neurons. The red areas represent values of density searched by the positioning algorithm (see Methods). **(D)** 3D spatial arrangement of motor neurons in a volume of about 0.5 × 10^7^ μm^3^ in the thoracic spinal cord region. We note different size populations allowing us to distinguish the motor neurons (enclosed in red circles) from other cells whose size is compatible with glia (enclosed in black squares). See Methods and Fig. 2 of SI.

**Figure 6:**
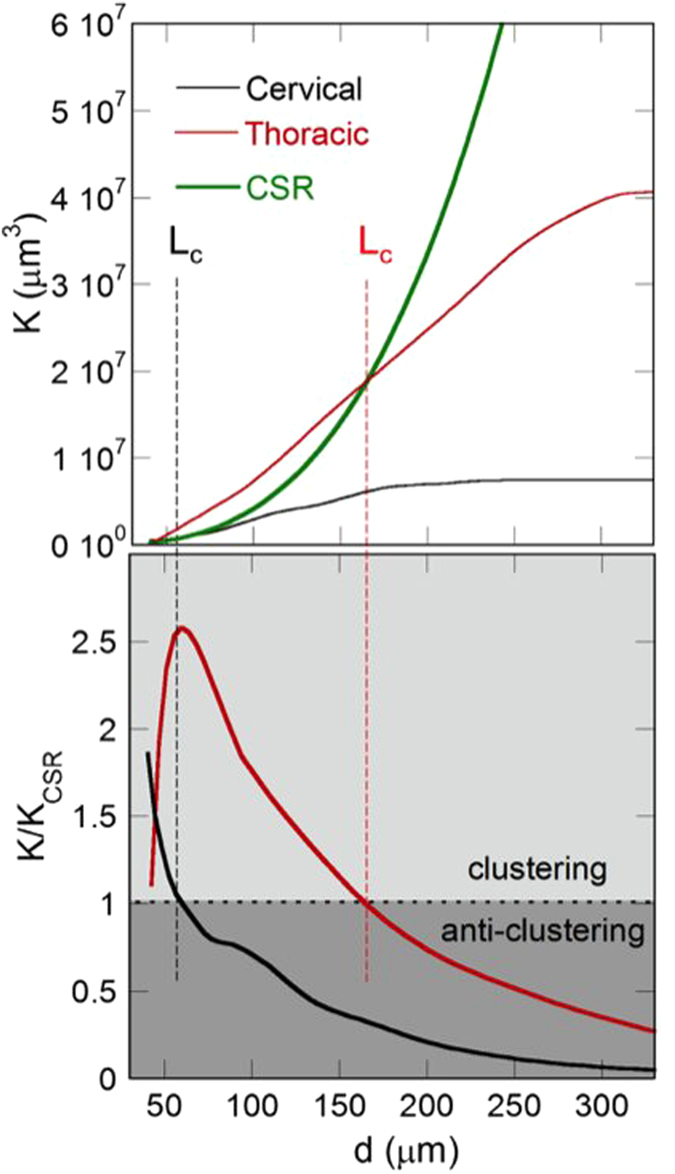
(Upper panel) K-Ripley function of the motor neurons calculated in a typical volume of (red line) thoracic and (black line) cervical region. The thick green lines represent the K-Ripley function of randomly located neurons. (Lower panel) K/K_CSR_ ratio, of the motor neurons calculated in the same volume in (red line) thoracic and (black line) cervical region. In the dark regions (ρ > 1) the neurons tend to clusterize on distances, *L*_*c*_, indicated by the dashed lines at 165 μm and 55 μm in the thoracic and cervical regions, respectively.

A quantitative spatial statistical analysis needs high-quality images at high spatial resolution and a large field of view, as those used here. The availability of these images allowed us to study the spinal cord morphology at a large scale (Fig. 1), as well as the neurons distribution at the grey/white matter interface (Fig. 2) and in intermediate grey matter up to the single neuron interaction (Figs 3,4). In Figs 1,2,3,4,5,6 we report the results obtained in the case of a representative healthy mouse; the quantitative analysis does not show significant differences among the four specimens measured. In particular, the healthy samples will serve as a reference for studies on mice affected with neurodegenerative diseases.

In Fig. 1A, a representative 3D reconstruction of a ~200 μm-thick volume (axial section) in the cervical region is reported. The striking contrast between white and gray matter (reversed colors in the figure) allows to image the neuronal cell bodies, appearing as white spots in the gray matter, and the nerve fibers surrounding the gray matter, as confirmed by immunohistochemical analysis of SMI-32 (Fig. 1B), a marker of motor neurons, and hematoxylin/eosin staining (Fig. 1C).

To study the morphology of the white and grey matter interface, we analyzed the reconstructed volume of the axial section of the ventral horn (about 1 mm thick) in the thoracic level (the neurons are rendered in yellow; the nerve fibers, in violet) reported in Fig. 2A. This region includes groups of cells that form motor nuclei in the Lamina IX^19^. Figure 2B is a zoom of the interface between gray and white matter. The submicrometric resolution of the image allows the identification of the axonal processes emerging from motor neurons and directing outside the spinal cord, towards the ventral nerve root filaments. Figure 2C shows the potentiality of the technique: the neuronal network is imaged in sagittal cross section, 500 μm thick volume relative to the selected Region Of Interest (ROI) in the ventral horn (red box in Fig. 2A).

The longitudinal distribution of white matter and the motor neurons pool (or motor nucleus) in the ventral horn are discernible^19^.

In Fig. 3A a single neuron connection in the longitudinal view, compatible with an axon fiber with an average diameter of about 5 μm is magnified. Figure 3B displays a zoom of a similar connection between two motor neuron cells in the axial cross section of the ventral horn. The dendrites of the cells and the axon hillock are imaged. To parallel the XPCmT imaged neuron, we reported in Fig. 3C an high magnification of a SMI-32 positive neuron.

Figure 4 shows a sagittal view of the tomographic volume section at the interface between the central region and the ventral horn. The bottom insets of the figure display magnified view of a region comprising several motor neurons (red box), with an average size of the order of 30–50 μm, and cells at the medial border of the motor nucleus (blue box), with an average size of 10–20 μm in diameter (the latter are compatible with interneurons). In addition, in both insets there are small cells (less of 7 μm in diameter) compatible with the glia cells.

### Statistical analysis

We investigated the spatial distribution of the motor neurons in the ventral horns at different levels of the spinal cord (cervical, thoracic and lumbar regions). Indeed, the volume and the shape of grey substance vary significantly on different transverse sections along the spinal cord.

On an anatomical basis, we selected a ROI in the ventral horn (Laminae 8/9-motor nucleus) that is rich in motor neurons (see i.e. Figs 2,3). Within the chosen ROI we distinguished the motor neurons from other cells (i.e. glia cells) on the basis of their typical size.

The different grey levels (colors) of the XPCmT image are proportional to the electronic densities of the different elements. The 3D reconstructed tomographic volume of a typical single motor neuron (Fig. 5A), allowed us to investigate the variation of the density distribution inside the neuron soma at different depths (Fig. 5B) by a virtual slicing. We can distinguish the more intense nuclear region, which corresponds to the red tails of the probability density function of the electronic density in motor neurons (see Fig. 5C). This allows us to locate each cell and to discriminate between different cells populations by their size (Fig. 5D), as described in the Methods section. In order to unequivocally validate our approach for differentiating, locating and positioning the motor neurons, we compared the obtained results on transverse 20 μm thick tomographic slabs with the quantitative analysis of the histological transverse section (20 μm thick), undergoing to immunohistochemical analysis of SMI-32, a motor neuron marker^20^ (see Supplementary Information and Fig. 1A,B).

**Figure 7:**
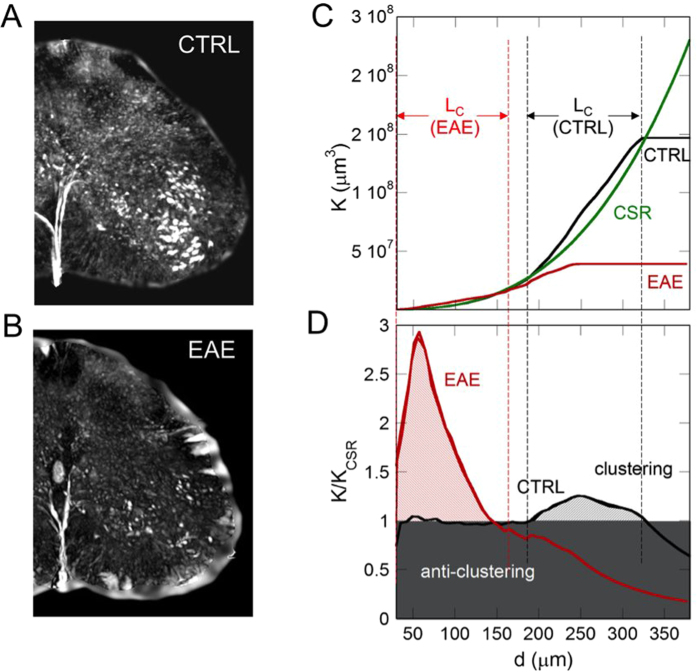
Phase contrast tomographic volume, 700 μm thick of (**A**) healthy (CTRL) and (**B**) EAE samples. (**C**) K-Ripley function of the motor neurons calculated in a typical volume of (red line) EAE and (black line) CTRL samples. The thick green lines represent the K-Ripley function of randomly located neurons. (**D**) K/K_CSR_ ratio, of the motor neurons calculated in the same volume of EAE (red line) and CTRL samples (black line). In the white region (ρ > 1) the neurons tend to clusterize within the range lengths, *L*_*c*_, indicated by the dashed lines at 145 μm and at [185–330] μm in EAE and CTRL samples, respectively.

After obtaining the position of each neuron in the 3D volume, we quantitatively analyzed the spatial distribution of the motor neurons. In particular, we used a 3D autocorrelation analysis to investigate the tendency of neurons to be either aggregated or dispersed. To this end, we calculated the K-Ripley function^21^ in the selected ROIs in the different spinal cord levels (Figs 6,7). The regularity in the neurons arrangement was quantified by Voronoi fluctuations^17^ (see Table 1), as described in Methods section.

In the healthy mice, the motor neurons clusterize with a different clustering distance (L_c_) at different levels of the spinal cord. Indeed, *L*_*c*_ = 55 μm, 165 μm and 330 μm at the cervical, thoracic and lumbar level, respectively. At the same time we have a clustering degree ρ = 0.05 at the cervical level, ρ = 0.20, at the thoracic level and ρ = 0.11 μm at the lumbar level. Also the Voronoi average volumes and fluctuations, change significantly in the three different SC levels (see Table 1).

The validity and potentiality of our global approach have been confirmed through its application to the case of a motor neuron disease model. In particular, we compared the results obtained for the healthy spinal cord with spinal cord affected with EAE in the lumbar region, where the motor neurons degeneration is evident^22^ (Fig. 7). In Fig. 7B, we report a reconstructed volume of 700 μm in thickness showing the degeneration and alteration of the motor neurons organization in mouse affected with EAE (in a pre-chronic phase, 5 days after the disease onset). An identical volume of the lumbar spinal cord of a healthy mouse is shown in Fig. 7A for comparison. We observed a significant decrease of the neuron density in the EAE sample, as confirmed in literature^23^ and by histological analysis of the pyknotic neurons shown in the Supplementary Fig. S2. At the same time the Voronoi average volume decreases, contrary to what would be expected for uniform regular spatial distributions. Moreover we measure i) the reduction of the clustering lengths, (*L*_*c*_ < 145 μm) and ii) the increase of the clustering degree, (ρ = 0.30) in the diseased sample. These results indicate that a local aggregation takes place in the motor neurons network during the EAE degenerative process.

## Discussion

The spinal cord is one of the most investigated nervous systems, formed by complex networks of motor neurons, interneurons, sensory afferents and motor efferent and descending and ascending tracts. We quantitatively studied the spinal cord architecture and the spatial distribution of the motor neurons, using high-resolution XPCmT. We visualized the 3D morphology of gray and white matter from the nerve fibers to the smaller neurons (Figs 1,2,3,4), on the entire sample and without any invasive sample preparation.

We also imaged the motor nucleus in the laminar arrangement at different spinal levels (see for example Fig. 2, showing a thick sagittal cross-section of the spinal cord at the thoracic level, where the motor columns are well-defined). The high spatial resolution of the technique enabled the study of the cyto- and dendro-architecture of the spinal motor nuclei in the ventral horn. In addition, we studied the relationship between the motor neurons and their spatial distribution in volumes of tissue at the cervical, at the thoracic and at the lumbar level, in the ventral horn of the healthy mice and in the lumbar level in the EAE-affected mice. We modelled their positioning in 3D space (see a representative example in Fig. 5). The electronic density distributions of the motor neurons in the different spinal cord levels, show a fatter tail, with an asymmetric behaviour characterized by a skewedness > 1^24^,^25^. It is well known how deviations from a normal distribution in the right tail are related with a more complex behaviour in several systems and processes^26^. In this case, the fatter tails suggest a complex morphology for the motor neurons.

Using the K Ripley function, we also analysed the spatial distribution of motor neurons, as well as their tendency to be aggregated, to be random or to be dispersed, (see the Methods) in three different regions of the spinal cord (cervical, dorsal and lumbar) in a volume of about 10^7^ μm^3^.

We observed that the motor neurons have a clear tendency to clusterize (Fig. 6) with different characteristic clustering lengths, *L*_*c*_ and different clustering degree, ρ, depending on the spinal cord region. In particular, the data obtained in the healthy case, reported in Table 1, reflect the intricate 3D arrangement of neurons.

By comparing the results obtained in the healthy mice with the results obtained in the EAE-affected mice in the same ROI in the lumbar region (Fig. 7), we observed that the motor neurons in the EAE become rarer but also more aggregated, i.e., they tend to be more “clumped”. This points out to a local aggregation of the decreased motor neurons during the degenerative process, suggesting the existence of structural holes in the neural network. The missing links due to these structural holes reduce the efficient spread of information, since they reduce the number of alternative information pathways in the network. At the same time, this local aggregative phenomenon increases the centrality of the surviving neurons, since there is a larger probability that information is exchanged through these locally clustered zones.

Finally, the quantitative results, reported in Table 1, clearly underline:

1. The different distribution of the grey matter in the ventral horn for the different regions. In fact, the quantity of grey substance, as well as its shape on transverse sections, markedly varies at different levels of the SC. In the thoracic region the amount of grey substance is small (also with respect to the surrounding white substance), while it is greatly increased in the cervical and lumbar enlargements^19^.
2. The sensitivity of the approach to detect and/or monitor neuropathological phenomena.

To summarize, in the present work we successfully characterized the neuronal microanatomy structure in *ex-vivo* healthy mouse sample, and we showed that the XRPCT, with the auxilium of quantitative metrics, has the potential for microstructural characterization of CNS diseases. Certainly, the study of the organization of the neuronal network is a crucial physiological issue^27^, for which quantitative structural measurements are a key step. Indeed, the quantitative estimation of the morphological and topological parameters, characterizing the neuronal microanatomy, could allow a better understanding of several neural degenerative processes.

The approach described in this paper has great potential. First of all, it overcomes the limitation of conventional 2D imaging, which offers incomplete spatial coverage and is based on destructive sample preparation, entailing possible data misinterpretation. At the same time, standard 3D computed tomography and MRI achieve insufficient spatial resolution (tens/hundreds of microns) and require aggressive staining and casting. Moreover, standard 3D computed tomography does not provide sufficient image contrast of soft low-density tissues, as it is the case of the spinal cord. On the contrary, we are able to obtain 3D images of any features of the white and grey matter in any region of the CNS with sub-micron spatial resolution and without any particular sample preparation. In addition, we can virtually slice the tomographic volume in the axial, sagittal and coronal projections, thus making it possible to study the 3D neuronal network with high resolution along all directions and at each depth level inside. This technique was also applied to the investigation of the brain, in particular to visualize amyloid plaques of a transgenic mouse model of Alzheimer’s disease. It allowed for quantifying several important structural parameters, such as the size of amyloid plaques and their regional density in 3D, over a volume encompassing the entire mouse brain^28^.

On the other hand, XPCmT also has some limitations, which are worth mentioning here. First, XPCmT does not permit the univocal identification of cells that can be easily achieved via marking in conventional histology. For this reason, we limited the application of our quantitative approach to the investigation of the motor nuclei, which are clearly resolved. Indeed, since the algorithm discriminates the different cell populations based on their anatomical position and size, the interneurons and the glia cells could be mixed up.

Moreover, even though in principle XPCmT can also be applied *in vivo*, nevertheless several limitations will be met in this case, in terms of spatial resolution, image quality and delivered dose.

In conclusion, beyond these limitations, the high spatial resolution, the achievable contrast, and the quantitative analyses with possible implementation make the described methodology sensitive to pathological changes. Therefore, in perspective, our approach could be applied to the preclinical investigation of the CNS pathologies. Indeed, this kind of approach permits to characterize the axonal and neuronal degenerative processes, providing valuable information for the assessment of therapeutic strategies and for the management of patients.

## Methods

### Sample Preparation

All experimental animal procedures were carried out in the IRCCS AOU San Martino–ISTAnimal Facility (Genoa, Italy), in the respect of the national current regulations regarding the protection of animals used for scientific purpose (D.Lgs 27/01/1992, n. 116). Research protocols have been evaluated and approved by the IRCCS AOU San Martino –IST Ethical Committee for animal experimentation (CSEA) as Animal use project n. 336 communicated to The Italian Ministry of Health, having regard to the article 7 of the D.Lgs 116/92.

Mice were purchased from Charles River, Calco, Italy and kept under controlled conditions (temperature: 22 °C; humidity: 40%) on a 12-h light/dark cycle with food and water ad libitum.

We studied the spinal cord of healthy adult male C57 Black mice (20–22 g, body weight) and EAE-affected mice. Female 6–8 week-old C57Bl/6 J mice (18–20 g) were immunized for EAE as described by subcutaneous injection at two sites in the flank with an emulsion of 200 μg myelin oligodendrocyte glycoprotein (MOG) peptide 3555 (Espikem) in incomplete Freund adjuvant (IFA; Difco) containing 300 g Mycobacterium tuberculosis (strain H37Ra; Difco). Mice were injected in the tail vein with 400 ng pertussis toxin (Sigma-Aldrich) immediately and 48 h after immunization. The mice were scored daily for clinical manifestations of EAE on a scale of 0–5^29^.

Mice were divided into two groups: one group composed by four healthy and three EAE-affected mice was used for XRPCT, and the other group composed by one sample for each category used for histology and immunohistochemistry analysis.

Group 1: sample preparation (for XSPCmT): Perfusion with saline solution

Mice were perfused transcardially with saline solution containing heparin (50 U/ml). Afterwards spinal cords were dissected out, fixed in 4% paraformaldehyde for 24 h, and then maintained in 70% alcohol until analysis. Before the XRPCT experiments mice underwent laminectomy and the spinal cord was taken off, dehydrated using graded ethanol and immersed in methyl salicylate for 24–48 h.

Group 2: Histology and immunohistochemistry

Mice were sacrificed and spinal cords were dissected out and immediately fixed for 24 h in ethyl alcohol (60%), acetic acid (10%) and chloroform (30%) and then embedding in paraffin for hematoxylin/eosin staining histology and for immunohistochemical analysis of SMI-32.

In particular, for hematoxylin/eosin staining histology after embedding in paraffin, spinal cord sections were cut at 20 μm and stained. For immunohistochemical analysis of SMI-32 after embedding in paraffin, 20 μm sections were first soaked in 3% hydrogen peroxide to block endogenous peroxidase activity and then incubated overnight with mouse monoclonal anti-SMI32 (1:1,000, Covance, Princeton, NJ). Slices were then incubated for 1 h with secondary biotinylated anti-mouse antibodies (1:200; Vector Laboratories, Burlingame, CA). 3,3-Diaminobenzidine tetrachloride was used for detection (ABC Elite kit; Vector Laboratories, Burlingame, CA). Control staining was performed without the primary antibody.

### Synchrotron Phase Contrast Tomography: Experimental approach and tomography reconstruction

The SXPCT has been performed on the samples of group 1. We measured the entire mouse spinal cord of the healthy and EAE-affected mice with XSPCmT (in free space propagation mode) without using contrast agent.

The experiment for healthy mice was carried out at TOMCAT beamline at the Swiss Light Source (SLS) in Villigen (Switzerland). The monochromatic incident X-ray energy was 17 keV and a CCD camera with a pixel size of 0.64 microns was set at a distance of 5 cm from the sample. The tomography has been acquired with 1601 projections covering a total angle range of 360°. The experiment on EAE-affected mice was instead performed at the ID17 beamline at the ESRF (France). The monochromatic incident X-ray energy was 30 keV. The sample was set at a distance of 2 m distance from the CCD camera having a pixel size of 3.5 μm. The phase retrieval algorithm has been applied to the projections of the tomographic scans using a modified version of the ANKAphase code^30^,^31^. The different electron densities of the tissues were rendered as grey-levels in the phase tomograms images. The image analysis and the image segmentation to independently display the different tissues, have been performed using both free software (Volview, ImageJ) and software developed in-home (Matlab routines).

### Statistical Analysis

We developed an algorithm to individuate and locate different cell population nuclei in a given reconstructed volume. We have selected Regions of interest (ROIs) in the ventral horn (rich in motor neurons) in different spinal cord levels, in a volume of about 10^7^ μm^3^. The neuronal intracellular electronic density distribution clearly shows a modulation, with the highest values reached in the nucleus (see Fig. 5).

The algorithm is based on the following steps: first, the probability density function (PDF) of the neurons image intensity was calculated. Then we selected the voxel intensity values higher than a threshold. In a first good approximation, the threshold is given by <I> + 2ΔI, where <I> and ΔI are the mean and the standard deviation of the intensity in a volume occupied by the neuronal cell respectively. These values, falling in the red tail of the PDFs (Fig. 5C), correspond to the neuronal cell volumes. Finally, we selected the volumes larger than 7 × 7 × 7 μm^3^ in order to avoid artifacts and other small cells such as glial cells (see Supplementary Fig. S3).

The 3D point patterns, given by the position of each neuron in the considered volume, have been quantitatively characterized by using 3D autocorrelation and Voronoi tessellation (Qhull code for Convex Hull, Delaunay Triangulation, Voronoi Diagram, and Halfspace Intersection about a Point, http://www.qhull.org) to study the spatial arrangement of neurons.

This method was validated with the histology^20^ (see Supplementary Information).

### The autocorrelation analysis

The autocorrelation analysis indicates if neuron positions show a tendency for clustering (aggregation), or for anti-clustering (dispersion)^32^. We performed the 3D spatial correlation analysis using the K-Ripley function. This function is the cumulative histogram of the average density of cells as a function of distance from the origin that is the well-known density recovery profile (DRP)^32^ In particular, the K function considers the expected number of cells within a spherical shell at a given distance from a cell^32^.

A limitation of the K-Ripley calculation is the edge correction term, due to the proportion of spherical volumes outside the sample boundaries. To overcome the limitation we correct for the relative edge effects^33^. We compared the sets of spatial patterns with the null hypothesis of complete spatial randomness (CSR), whose theoretical 3D K-function is given by:


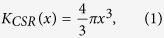


where *x* represents the range of distances, from 0 to the smaller size of the examined volume. In this way we could define a clustering degree on a characteristic length, Δ*x*, (representing a normalised measure of clustering/dispersion) as:


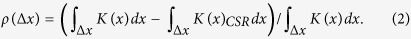


Thus, we have ρ > 0 for clustering (aggregation) arrangement and ρ < 0 for dispersing arrangement. We can also define a characteristic clustering length, Δ*x* = *L*_*c*_, indicating the spatial range, *K(x*) > *K*_*CSR*_(*x*), above which neurons are aggregated.

### The 3D Voronoi tessellation

3D Voronoi tessellation analysis characterises the regularity of neural cells arrangement. It consists of a set of tetrahedral domains built by neurons natural neighbors^32^ 3D Voronoi domains have been computed using standard routines within Qhull (Qhull code for Convex Hull, Delaunay Triangulation, Voronoi Diagram, and Halfspace Intersection about a Point, http://www.qhull.org). The regularity of neurons arrangement is described by the Voronoi volumes fluctuations, *dV*_*v*_, given by *dV*_*v*_ = Δ*V*_*v *_*/V*_*v*_ where Δ*V*_*v*_ and *V*_*v*_ are the standard deviation and the average values, respectively, of the Voronoi tetrahedral volumes.

## Additional Information

**How to cite this article**: Bukreeva, I. *et al*. Quantitative 3D investigation of Neuronal network in mouse spinal cord model. *Sci. Rep.* **7**, 41054; doi: 10.1038/srep41054 (2017).

**Publisher's note:** Springer Nature remains neutral with regard to jurisdictional claims in published maps and institutional affiliations.

## Supplementary Material

Supplementary Information

## Figures and Tables

**Table 1:** Neuron density, Voronoi volume and Voronoi fluctuations in a typical volume of about 10^7 ^μm^3^ of the cervical, thoracic and lumbar regions.

MOTOR NEURONS					
Spinal cord levels	Neuron density (mm^−3^)	Average Voronoi Volume (μm^3^)	Voronoi Volume fluctuation	L_c_ (μm)	ρ(L_c_)
Cervical	(3.0 ± 0.5) × 10^3^	(2.0 ± 0.2) × 10^5^	1.7 ± 0.2	55 ± 5	0.05 ± 0.01
Thoracic	(2.0 ± 0.4) × 10^3^	(5.0 ± 0.6) × 10^5^	2.3 ± 0.3	165 ± 18	0.20 ± 0.04
Lombar CTRL	(5.0 ± 0.6) × 10^3^	(7.5 ± 0.7) × 10^5^	2.5 ± 0.4	330 ± 32	0.11 ± 0.03
Lombar-EAE	(1.0 ± 0.2) × 10^3^	(2.0 ± 0.3) × 10^5^	1.6 ± 0.2	140 ± 20	0.30 ± 0.05
