# Supplementary material for: Quantitative 3D investigation of Neuronal network in mouse spinal cord model
Source: Sci Rep. 2017 Jan 23;7:41054. doi: 10.1038/srep41054 (PMC5253662; doi:10.1038/srep41054)
Supplement: Supplementary Information [file srep41054-s1.pdf]

Quantitative 3D investigation of Neuronal network in mouse spinal cord model

I. Bukreeva [1&], G. Campi [2&], M. Fratini [1,3&], R. Spanò[4], D. Bucci[5], G. Battaglia[5], F. Giove[3,6], A. Bravin[7], A. Uccelli[8], C. Venturi[8], M. Mastrogiacomo[4] & A. Cedola[1*]

1. Institute of Nanotechnology-CNR c/o Physics Department at 'Sapienza' University, Piazzale Aldo Moro 2, 00185 Rome, Italy.
2. Institute of Crystallography-CNR, 00015 Monterotondo, Rome, Italy.
3. Museo Storico della Fisica e Centro Studi e Ricerche Enrico Fermi, Piazza del Viminale 1, 00184 Roma, Italy,
4. Department of Experimental Medicine, University of Genova & AUO San Martino - IST Istituto Nazionale per la Ricerca sul Cancro,
Largo R. Benzi 10, 16132 Genova, Italy.
5. I.R.C.C.S. Neuromed, Località Camerelle, 86077 Pozzilli, Italy.
6. Fondazione Santa Lucia I.R.C.C.S., Via Ardeatina 306, 00179 Roma, Italy
7. European Synchrotron Radiation Facility, 71 Avenue des Martyrs, 38043 Grenoble, Cedex France
8 *University of Genova DINOGMI Largo Daneo, 3 IT-16132 Genova*

& Equal contribution
*Corresponding author: alessia.cedola@cnr.it

Supplementary Information

To confirm the validity of our approach we compare the results obtained by applying our algorithm on a tomographic reconstructed slice (20 micron thick) with the results obtained with a standard quantitative analysis on a histological section (20 micron thick), treated with a motor neurons marker (immunohistochemical analysis of SMI-32). As shown in the fig. S1 our approach locates the neurons in the tomographic image in the same region where they appear in the histology. The final neuron density in the histology, following the counting procedure described in Battaglia et al. (2015)[20], is about 6 $x10^3$ in the healthy mouse. In the x-ray tomography, where our approach has been applied, we found out 5 $x10^3$.

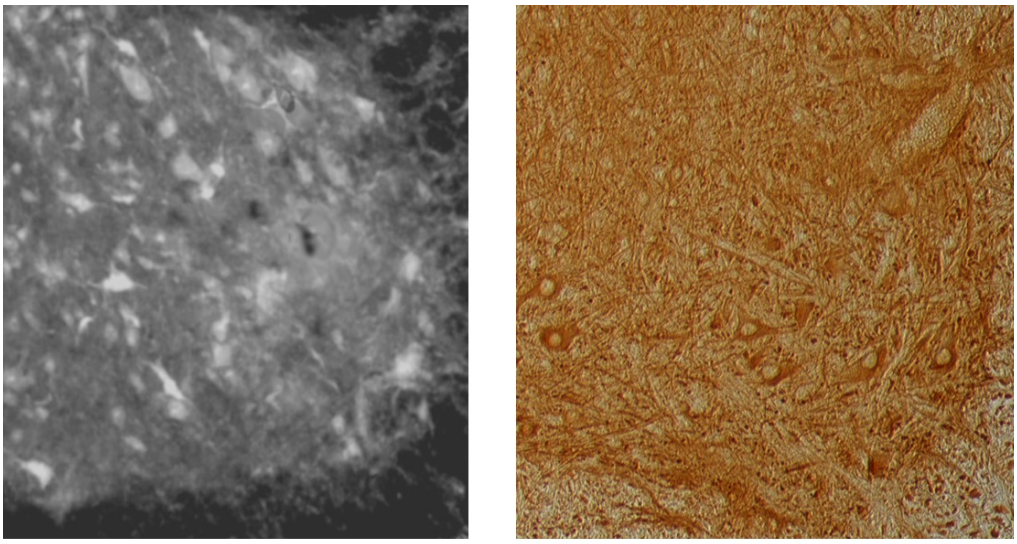

A B

Figure S1: A) Tomographic reconstructed slice of the healthy mouse spinal cord in the ventral horn of the cervical region (20 micron thick). **B)** Immunohistochemical analysis of SMI-32, a marker of motor neurons, of a representative healthy mouse spinal cord in the ventral horn of the cervical region, where was applied the statistical analysis to

distinguish the motor neurons to confirm the potentially of our approach for the 3D reconstruction of imaged spinal cord**.**

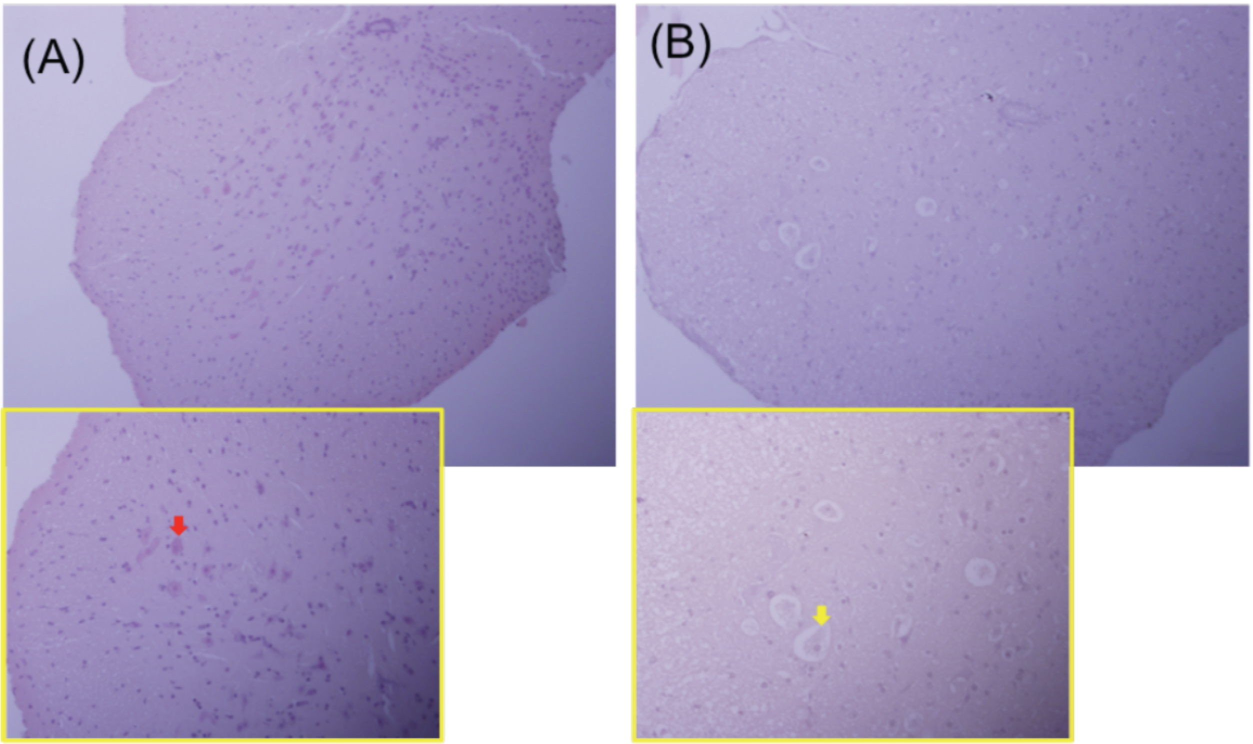

Figure S2: Population of cells in the histological sections (coloured with H/E) of the lumbar region in healthy **(A)** and EAE **(B)** mouse. The red arrow indicates neurons while yellow arrow indicates neurons with a pyknotic nucleus.

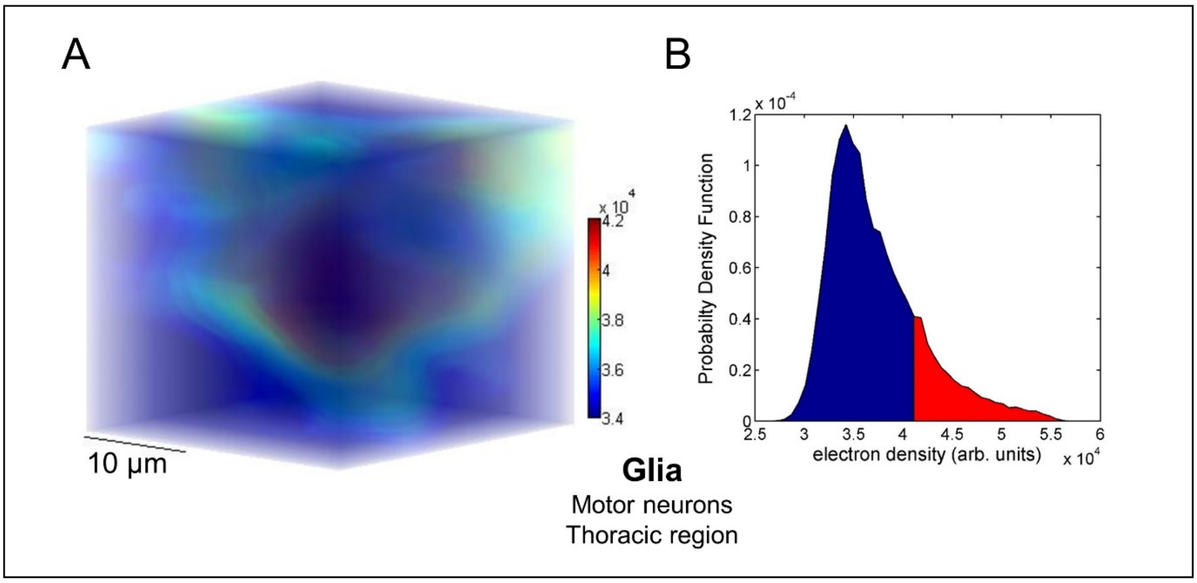

Figure S3 (A) 3D electronic density of a typical single glia in the thoracic region. **(B)** The probability density distribution of the glia. The red areas represent values of density searched by the positioning algorithm.
